# The Impact of Medication Synchronization on Proportion of Days Covered within the Pediatric Setting

**DOI:** 10.1097/pq9.0000000000000657

**Published:** 2023-05-22

**Authors:** Brooke E. Maletic, Alex Swick, Leanne Murray, Mahmoud Abdel-Rasoul, Ashley Braughton, Kayla Petkus

**Affiliations:** From the * Department of Pharmacy, Nationwide Children’s Hospital, Columbus, Ohio; § Biostatistics Resource at Nationwide Children’s Hospital (BRANCH, Center for Biostatistics), Columbus, Ohio; ‡ Department of Pharmacy, Information Systems, Nationwide Children’s Hospital, Columbus, Ohio; † Complex Healthcare Clinic, Nationwide Children’s Hospital, Columbus, Ohio.

## Abstract

**Introduction:**

Poor adherence to medication regimens accounts for the substantial worsening of disease, death, and increased healthcare costs of approximately $100 billion annually in the United States. Patients participating in medication synchronization had 3.4 to 6.1 times increased odds of adherence, depending on the drug class. Abundant literature supports medication synchronization within the adult population. This IRB-exempt, prospective quality improvement project is an example of implementing and assessing medication synchronization inclusive of the pediatric setting.

**Methods:**

This study is a single-center, prospective, quality improvement project for patients seen at Nationwide Children’s Hospital (NCH) Complex Care Clinic that also fill prescriptions at NCH Outpatient Pharmacies. The project assessed patient medication adherence using the Proportion of Days Covered and the number of trips to the pharmacy 90 days before and 90 days postimplementation. We also assessed patient and pharmacy staff satisfaction 3 months after project implementation.

**Results:**

There was a statistically significant increase in the number of days covered for patients 90 days postimplementation compared to 90 days before implementation (Difference: 3.60; 95% confidence interval: 1.87, 5.33; *P* = 0.001). Additionally, there was a statistically significant decrease in pharmacy trips pre- and postimplementation (Difference: 2.17; 95% confidence interval: 1.26, 3.07; *P* < 0.001). Overall, pharmacy staff and patients reported satisfaction with the service.

**Conclusions:**

Implementing a medication synchronization service improved medication adherence and decreased trips to the pharmacy within the pediatric population.

## INTRODUCTION

### Available Knowledge/Rationale

Poor adherence to medication regimens accounts for the substantial worsening of disease, death, and increased healthcare costs of approximately $100 billion annually in the United States.^1^ Many barriers contribute to poor medication adherence. Common reasons patients give for not taking medications as prescribed include forgetfulness, other priorities, the decision to omit doses, lack of information, and emotional factors.^1^ Complex medication regimens, cost of medications, and numerous trips to the pharmacy can also increase the patient burden and decrease medication adherence. Some pharmacies have implemented medication synchronization services (Med Sync) to combat these challenges and improve patient care. The number of patients enrolled in Med Sync services from 2014 to 2017 is estimated to have increased from 355,000 to 3.5 million.^2^ Patients participating in Med Sync had 3.4 to 6.1 times increased odds of adherence, depending on the drug class.^3^ Abundant literature supports Med Sync within the adult population. This IRB-exempt, prospective quality improvement project is an example of implementing and assessing Med Sync, inclusive of the pediatric setting.

### Problem Description

Nationwide Children’s Hospital, located in Columbus, Ohio, is the second largest free-standing pediatric hospital in the United States, with outpatient pharmacy services that filled over 264,000 prescriptions in 2021. In June 2020, as part of the COVID-19 pandemic efforts to decrease contact between staff and patients, our pharmacy offered curbside pick-up of medications. Patients and their families remained outside, and a pharmacy staff member brought the prescriptions to the identified patient. Operational challenges and a decrease in utilization led to the discontinuation of the service after approximately one year. The pharmacy staff reviewed data to identify utilizers of this service. They found that our patients with complex care conditions and their caregivers were the most frequent utilizers and made multiple weekly trips to the pharmacy. With this observation, we recognized that our pharmacy had an opportunity to improve the quality of care for our patients. This article outlines our efforts to initiate a pilot Med Sync service at our institution to improve medication adherence while decreasing the burden on our families.

### Specific Aims

The pharmacy department staff care for our patients by advocating for medication access, serving patients with the highest quality and safest patient care, and providing safe and effective medication therapy to achieve the best outcomes. The purpose of this project aligned with the department’s mission to improve medication adherence, improve medication access, decrease visits to the pharmacy, and positively impact patient and staff satisfaction. Our primary objective, to assess medication adherence before and after implementing the Med Sync service, was measured using the proportion of days covered (PDC) as our metric. PDC is a percentage calculated by dividing the number of days covered by the number of days in that period. Secondary objectives include assessing the number of trips to the pharmacy before and after implementation and the satisfaction of patients and pharmacy staff after implementation.

## METHODS

### Context

Nationwide Children’s Hospital outpatient pharmacy services consist of four locations. Three pharmacies on the main hospital campus include the Blue, Orange, and Specialty Pharmacy. The Yellow Pharmacy is at an offsite ambulatory care center. Nationwide Children’s Hospital patients can fill prescriptions at any outpatient pharmacy. Enrollment in Specialty Pharmacy services requires additional criteria. Patients commonly utilize the Orange and Blue Pharmacies for prescription fills due to their proximity to clinics on the main campus, such as the Complex Health Care Clinic (CHC).

The CHC is a multidisciplinary clinic caring for children with medical complexity. This clinic serves children with neurologic conditions resulting in loss of function and physical disability, chronic medical conditions involving multiple organ systems, and medically fragile children that require many healthcare resources with frequent unplanned hospital admissions or emergency department visits. These patients often have extensive medication lists, resulting in caregivers making multiple weekly trips to the pharmacy. We targeted these patients for our work to best pilot a Med Sync service. A chart review of 615 CHC patients who fill prescriptions at Nationwide Children’s outpatient pharmacies determined which patients met the following inclusion criteria: four or more chronic medications, fill at NCH Outpatient pharmacy, and have prescription insurance coverage through Ohio Medicaid or Medicaid Managed Care plan. Ohio Medicaid and Medicaid Managed Care plans cover most CHC patients, allowing consistent billing among all patients versus varying commercial plan requirements. Patients were recruited via a patient portal message within the patient’s medical record or contacted via phone during the 2-week enrollment window. This pilot service was introduced to the families and children who met inclusion criteria with a brief, scripted explanation of the Med Sync service and the opportunity to participate within the 2-week enrollment window. This introduction was carried out by the resident and pharmacist embedded in the clinic. Both patient and individual prescriptions are enrolled. We established this defined enrollment period to create a successful pilot service and keep it in line with our project timeline.

The outpatient pharmacies utilize dispensing software integrated fully with the patient’s electronic medical record (Epic Systems, Verona, Wis.). Med Sync is an application within the electronic medical record with features to enroll prescriptions in Med Sync and suggests a synchronization date based on prior dispenses. An indicator specific to Med Sync is visible in the patient chart and on each enrolled prescription. From this, staff can recognize enrolled prescriptions to ensure all synced prescriptions are filled simultaneously. The indicator of an enrolled prescription is carried forward when a new prescription is sent for an enrolled medication, reducing the risk of it becoming accidentally unenrolled. Additionally, medications can be excluded by rules defined by pharmacy management, preventing staff from enrolling those prescriptions in Med Sync.

### Intervention

Implementation of Med Sync required foundational steps. First, we established a project team to lead this service in August 2021. The project team consisted of the Outpatient Pharmacy Manager, Outpatient Pharmacy Technician Supervisor, Information Systems Pharmacist, a pharmacist embedded in CHC, and a pharmacy resident. The project team met weekly to discuss and implement changes for improvement.

The project team determined how to optimally utilize the dispensing software functionality to ensure a smooth transition once the service was implemented. First, we developed our service workflow and analyzed how to integrate it into the standard pharmacy workflow. Second, we created a reference guide and reviewed the service workflow at two consecutive outpatient pharmacy staff monthly meetings.

The service workflow consists of two parts. First, aligning prescription fills to be due on the same date, and the second is providing refill coordination for subsequent fills. To support the workflow, the team created two reports for this service: one to find enrolled patients with medication refills due and one to find enrolled prescriptions with no refills remaining.

**Fig. 1.**
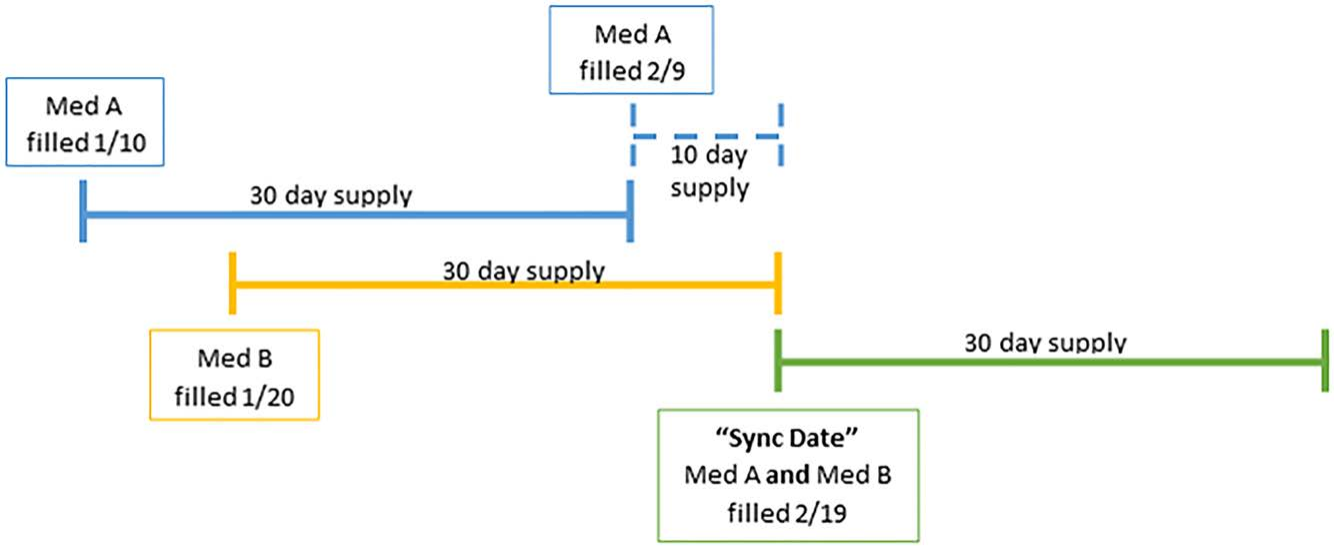
A patient is on two prescription medications, med A and med B, and the patient picked up these medications on two different days. On 1/10, we dispensed a 30-day supply of med A; on 1/20, we dispensed a 30-day supply of med B. If the future fill date, or sync date, were selected for the next time med B would need to be filled (2/19), then the dispensing software would calculate that a 10-day “short fill” of med A would need to be given to the patient on 2/9 for them to have enough at home. Then, on 2/19, we fill a 30-day supply of medications A and B for the patient and successfully synchronized the medications.

The pharmacy dispensing software functionality helped complete the first step. A patient-specific synchronization date (sync date) was determined based on when medications were best aligned. Certain medications require special consideration based on package size or regulatory restrictions. For example, software functionality excludes benzodiazepines because they cannot be included in a Med Sync program for Ohio Medicaid recipients.^4^ The software calculated medications on hand when the sync dates were determined. This calculation estimates medication remaining at home based on prior dispenses and is used to help determine what quantity is needed to reach a future sync date (Fig. 1). Next, the patient’s “short fills” for enrolled medications are filled to have sufficient medication until their sync date. A short fill consists of partially filling a prescription for a shorter day supply to align with the sync date. The full-day supply will be dispensed with all synchronized medications during the next fill.

The second step in our workflow provided ongoing refill support. A daily report identified enrolled prescriptions due to be filled in the range of 5 days in advance to 2 days past due. We processed prescriptions through the dispensing software system identified by the generated report. This report allowed proactive problem-solving, including prescription refill requests, insufficient inventory, and insurance rejections. The patient or caregiver was contacted to review the medication list, fill any as-needed medications upon request, and schedule a pick-up date. Prescriptions were filled and verified as part of the normal pharmacy workflow for a single dispense date.

### Study of the Interventions and Measures

The metric selected to measure medication adherence was the PDC. PDC has been incorporated within the US Centers for Medicare and Medicaid Services plan ratings and endorsed by the Pharmacy Quality Alliance as its recommended measure of adherence.^5–7^ PDC was calculated from prescription dispensing history data from the pharmacy dispensing software using the standard calculation method (number of days a patient has medication supply divided by the total number of days in the study period). PDC was calculated per prescription 90 days before and 90 days postintervention.

The number of pharmacy visits for each enrolled patient was determined by counting each unique date in the patient’s dispense history at Nationwide Children’s outpatient pharmacies. The data included dispenses for prescriptions not enrolled in Med Sync. This assessment was performed 90 days before the implementation of Med Sync (September 1, 2021, to December 1, 2021) and compared to 90 days after the enrollment period (December 17, 2021, to March 17, 2021).

**Fig. 2.**
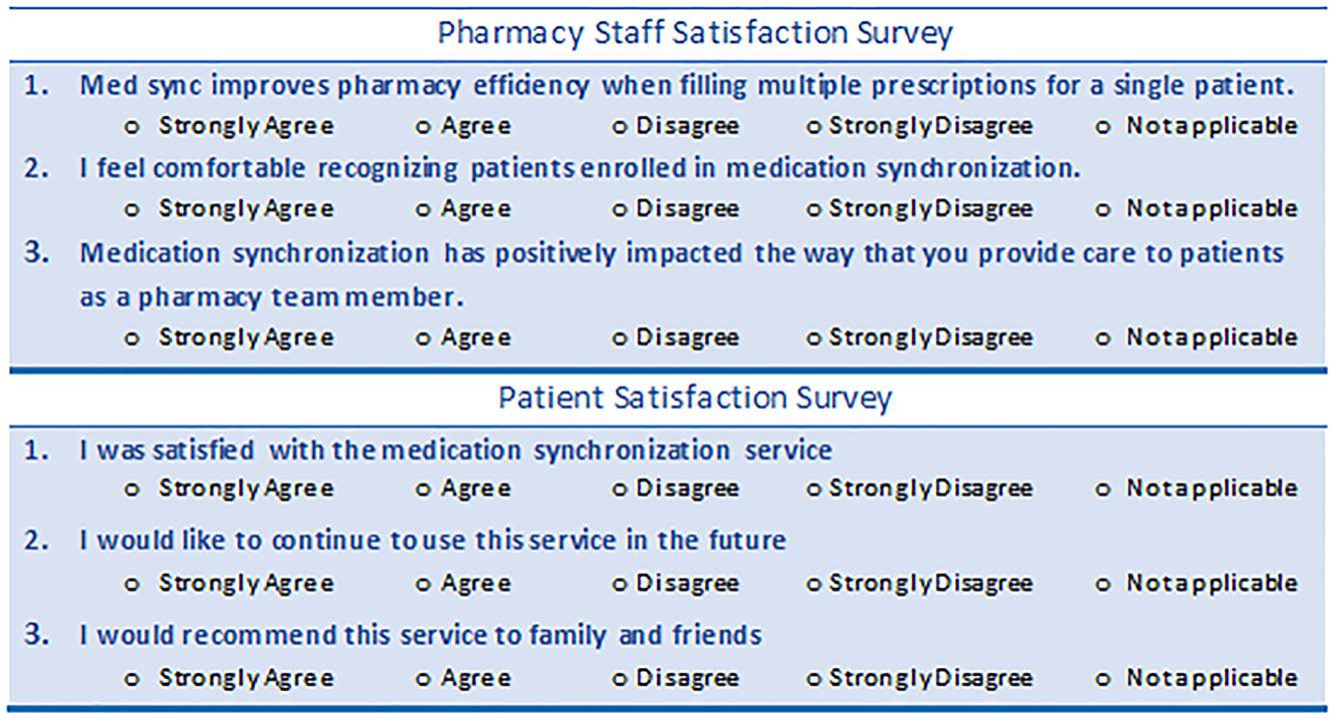
Pharmacy staff and patient satisfaction survey.

Outpatient pharmacy staff satisfaction was assessed via an electronic survey in the third month after implementation. The initial survey email was distributed 1 week before the survey closing, with two reminder emails. The survey consisted of three questions with responses on a Likert-type scale (Fig. 2). In addition, a project team member completed the patient satisfaction survey over the phone. Three contact attempts were made to collect information. This survey also consisted of three questions, with responses on a Likert-type scale (Fig. 2).

### Analysis

Summary statistics are reported as median [interquartile range (IQR)] or mean [95% confidence interval (CI)] where relevant for continuous variables. Categorical variables are reported as percentages. The average PDC percentages and the difference between study periods were tested using a linear mixed effects model with random intercepts to account for the correlation of within-patient repeated observations. Demographic and clinical variables, including gender, age, insurance type, race, and the number of chronic medications (**see table, Supplemental Digital Content 1**, http://links.lww.com/PQ9/A485), were assessed for association with PDC and potential confounding of the relationship between PDC and/or effect modification of the intervention. These variables were ultimately excluded from the final model and determined not to be confounders, effect modifiers, or PDC-associated. The number of trips to the pharmacy was compared between study periods using a paired t-test. Hypothesis testing was two-sided at a 5% type I error rate (alpha = 0.05). Statistical analyses were conducted using SAS version 9.4 (SAS Institute, Cary, N.C.).

### Ethical Considerations

This quality improvement project does not qualify as human subject research. Therefore, it did not require review and approval by the Nationwide Children’s Hospital institutional review board.

## RESULTS

We completed a chart review of 615 patients seen at Complex Care Clinic. The 215 patients that met inclusion criteria were contacted via patient portal message or phone. Of the patients contacted, 42 opted to enroll in this new service within the enrollment window. Our population had a predominantly white race (52.4%) and male sex (69.0%). The median age for our population was 10.5 [IQR: 8–17] years, with a median number of chronic prescriptions of 7 [IQR: 5–10].

**Fig. 3.**
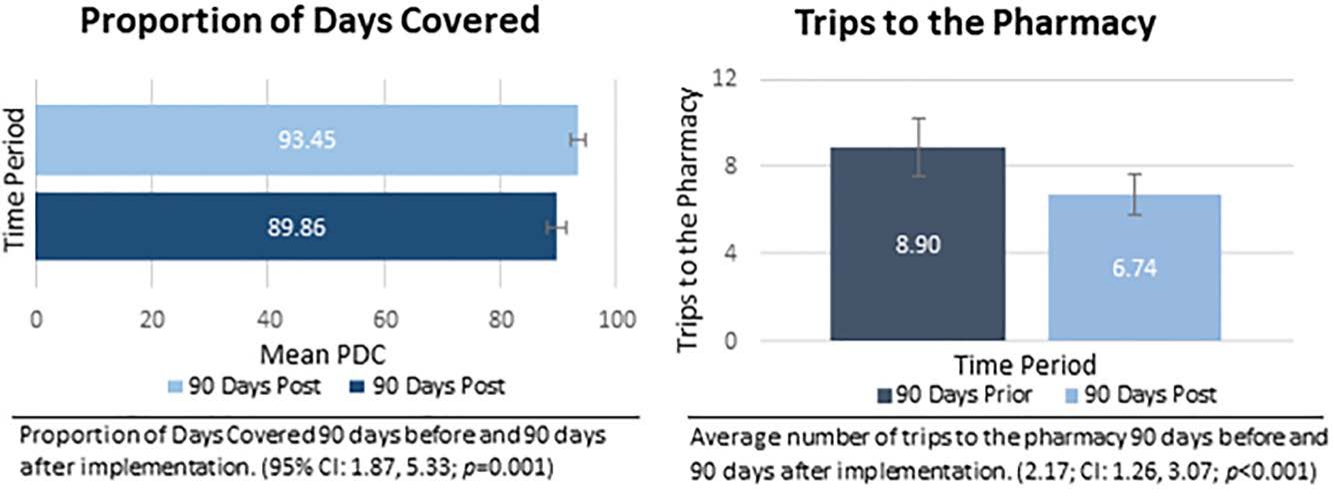
The primary objective, PDC, and the secondary objective, trips to the pharmacy.

The primary objective metric results can be found in Figure 3. Using a linear mixed model, we found that summative data showed a mean PDC of 89.86% (95% CI: 88.19, 91.52) for the 90 days before enrollment and 93.45% (95% CI: 92.18, 94.72) for the 90 days after enrollment (Difference: 3.60; 95% CI: 1.87, 5.33; *p* = 0.001). The secondary objective result, the average number of trips to the pharmacy, is highlighted in Figure 3. Before the implementation of Med Sync, the average number of trips to the pharmacy in 90 days was 8.90 (95% CI: 7.59, 10.22). However, the average number of trips to the pharmacy in 90 days decreased to 6.74 (95% CI: 5.81, 7.66) trips (Difference: 2.17; 95% CI 1.26, 3.07; *P* < 0.001) postimplementation of Med Sync.

**Fig. 4.**
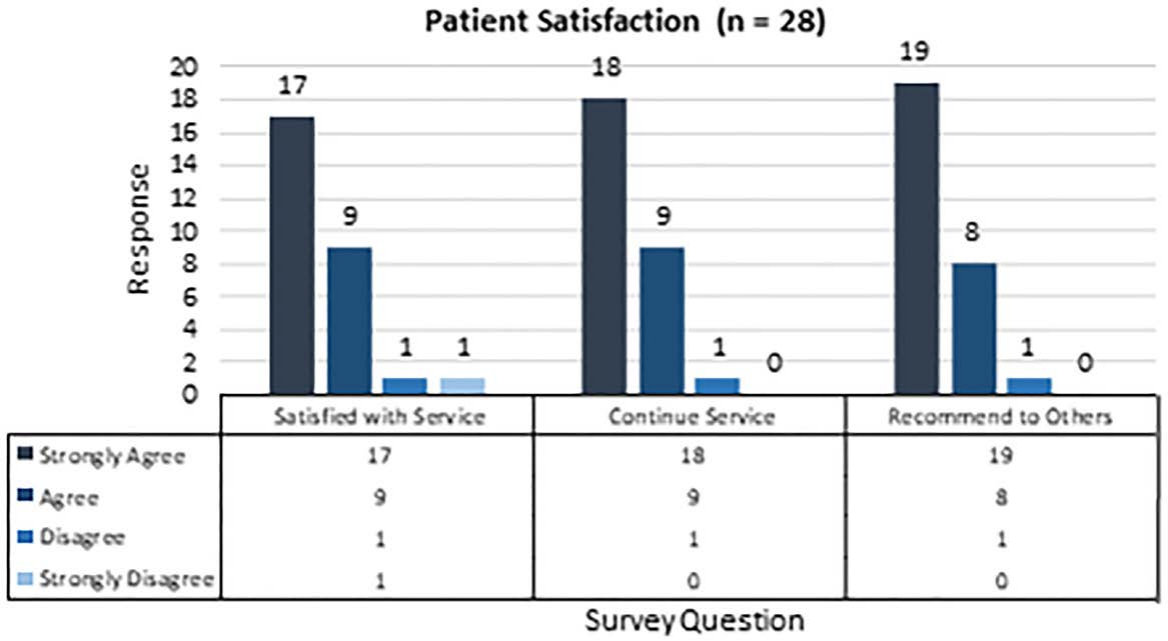
Twenty-eight patient satisfaction surveys were successfully collected.

**Fig. 5.**
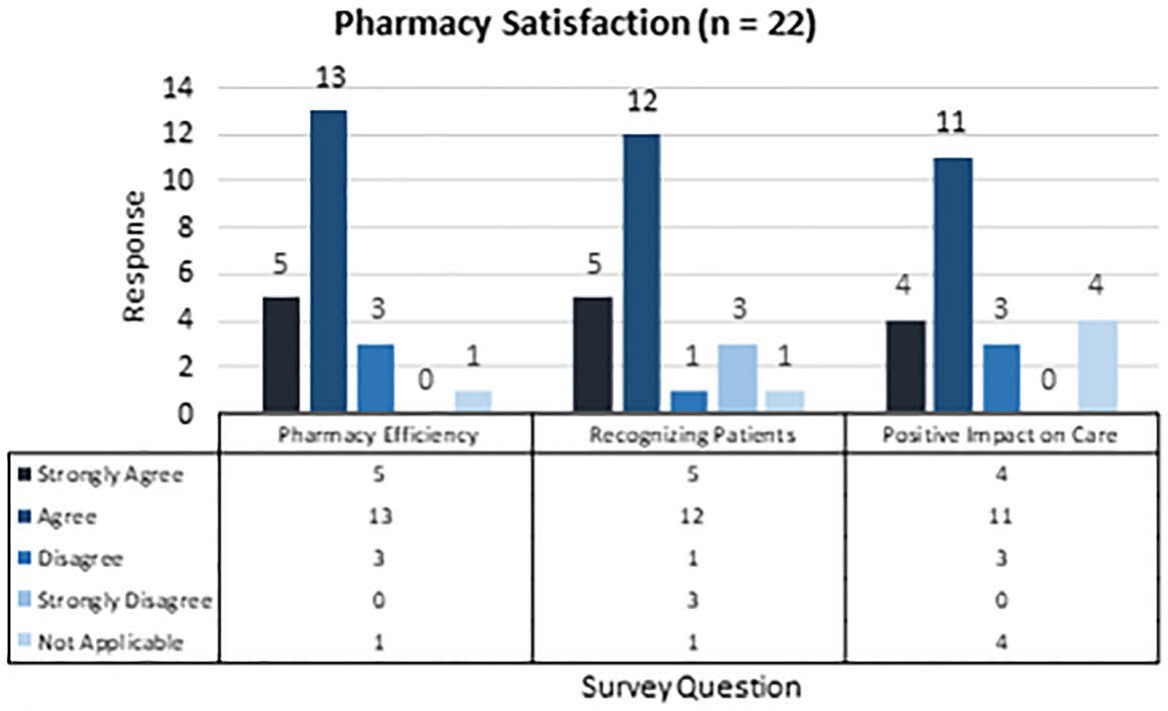
Twenty-two staff members completed the pharmacy staff satisfaction survey.

Patient and pharmacy satisfaction surveys are shown in Figures 4 and 5, respectively. Overall, patients agreed or strongly agreed to be satisfied with the new service, and 96% of patients wanted to continue this service and would recommend it to family and friends. Patients and caregivers provided additional comments, including feeling the service has been very helpful for them and that they could better manage their child’s medications. Eighty-two percent of pharmacy staff felt the pharmacy is more efficient when filling all medications at once than with numerous fills for one patient. Seventy-seven percent of staff felt comfortable recognizing patients enrolled in the service, and 68% felt the service positively impacted their patient care. The survey results show that staff members felt the service benefits patients but provided additional feedback that they would prefer to receive more education on its functionality.

## DISCUSSION

Despite the limited timeframe assessed, there was a statistically significant improvement in the mean PDC for our patient population. This finding suggests improvement in medication adherence throughout the assessment period. The Pharmacy Quality Alliance has defined a PDC of greater than 80% to have the most likelihood of clinical benefit.^8^ Mean PDC for our patient population was 89.86% for the preimplementation period making it difficult to infer the clinical significance of our intervention. In addition, it is unknown whether there is clinical significance with incremental increases to PDC already greater than 80% at baseline.

The number of trips to the pharmacy decreased significantly from approximately three trips per month to two trips per month. Although we hoped to see that number decrease to one trip, many factors out of our control can influence this measure. These factors include additional care encounters for acute or scheduled visits and new medication initiation or adjustments that might require a trip to the pharmacy outside the scheduled medication pick-up. In addition, this number included any “short fills” during the initial alignment of medications. Therefore, we hope this number will continue to trend downward as this service develops.

### Limitations

Despite statistically significant improvement in our primary objective, there were limitations throughout the project. Inclusion depended on timely caregiver response to our enrollment invitation for this service, so it was likely that participants were highly engaged before our intervention. The population’s high mean preimplementation PDC supports this engagement. Caregiver engagement may have also impacted the survey response rate. Additionally, completing the patient survey over the phone may have led to more positive feedback than if it had been administered electronically.

PDC as a measurement of medication adherence comes with its limitations. First, the PDC is a surrogate measure, and we cannot confirm the patient is taking medication as prescribed. Second, medication adherence is multifactorial, and our intervention did not target all potential barriers. Third, PDC does not account for hospitalizations during an assessment period, resulting in lower measures due to gaps in fill history. Fourth, most studies evaluate sustained improvement in PDC throughout 1–2 years. The short project timeline makes it challenging to show the clinical impact of improved medication adherence due to the intervention.

Last, the size and characteristics of our patient population introduced some limitations. These intentional limitations kept enrollment confined to a small group of patients from CHC with the same prescription payers to ensure the pharmacy team could operationalize this service. In addition, restrictions from other payers could limit the ability to extrapolate the results outside of Medicaid Managed Care or Ohio Medicaid.

## CONCLUSION

We report the first pilot of a Med Sync service and assessment conducted in the pediatric setting. Our results demonstrate a place for Med Sync services within the pediatric population with a positive impact on medication adherence, patient satisfaction, and decreased trips to the pharmacy.

### Future Directions

**Fig. 6.**
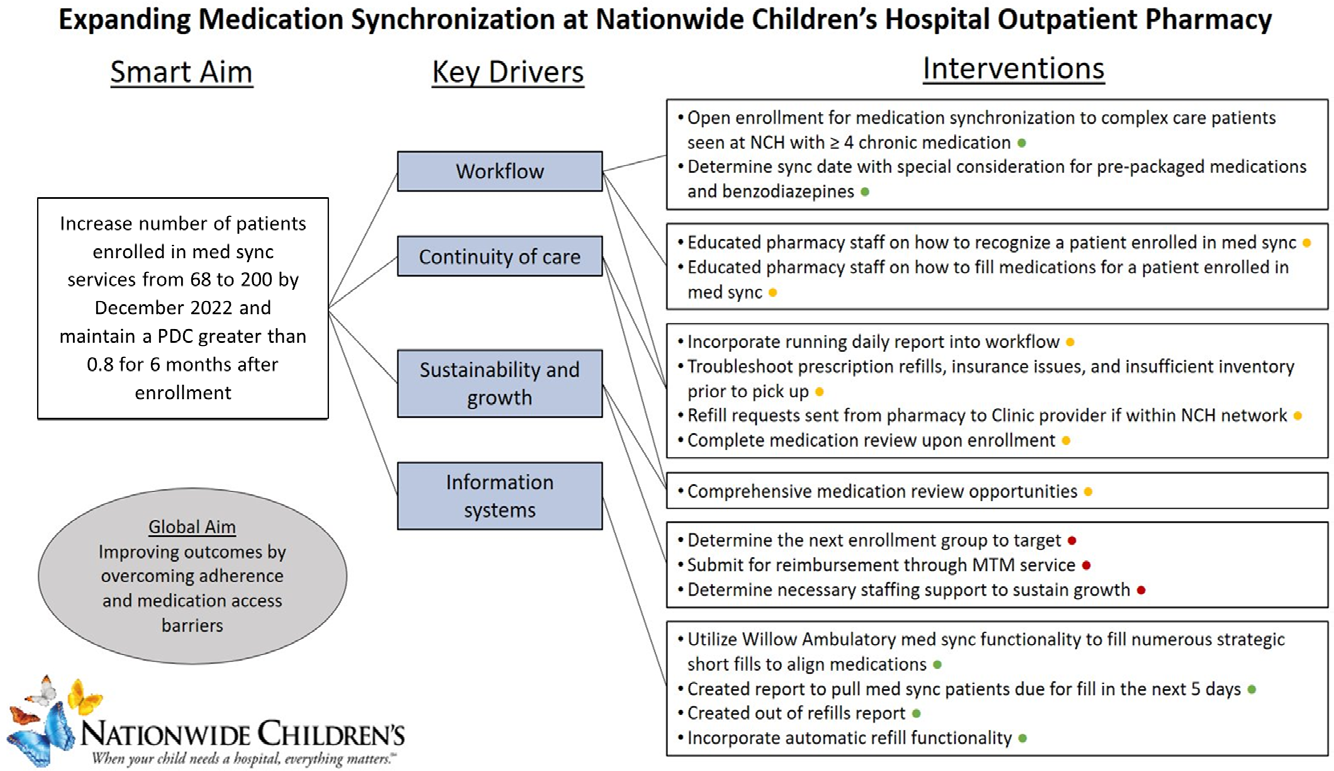
Key driver diagram for medication synchronization service.

Figure 6 outlines key improvement areas and interventions for the project moving forward. Future directions for this service include expansion beyond patients seen in Complex Health Care, expansion to patients with any insurance coverage, and targeting patients with lower PDC at baseline. In addition to changes to eligible patients, we will incorporate Medication Therapy Management services, such as comprehensive medication reviews, within the pharmacy workflow. Finally, we will continue following data as the service is expanded to new patients and for ongoing sustainment of PDC and trips to the pharmacy in current pilot patients.

## ACKNOWLEDGMENTS

Assistance with this study: Mahmoud Abdel-Rasoul.

## DISCLOSURE

The authors have no financial interest to declare in relation to the content of this article.

## Supplementary Material


