# Supplementary material for: The Impact of Medication Synchronization on Proportion of Days Covered within the Pediatric Setting
Source: Pediatr Qual Saf. 2023 May 22;8(3):e657. doi: 10.1097/pq9.0000000000000657 (PMC10990386; doi:10.1097/pq9.0000000000000657)
Supplement: Supplementary file 1 [file pqs-8-e657-s001.pdf]

Patient Demographics	
**Sex (n=42)**	
**Male**	69% (29)
**Female**	31% (13)
**Race (n=42)**	
**African American**	42.8% (18)
**Bi-Racial**	2.4% (1)
**White**	52.4% (22)
**Latino/Hispanic**	2.4% (1)
**Ethnicity (n=42)**	
**American**	92.8% (37)
**Spanish**	2.4% (1)
**Arabic**	2.4% (1)
**African**	2.4% (1)
	Median (range)
**Age (n=42)**	10.5 (3-32)
**Chronic Medications (n=42)**	7 (4-16)

Table 1. Patient demographics
